# Supplementary figures and images for: The up-regulation of TGF-β1 by miRNA-132-3p/WT1 is involved in inducing leukemia cells to differentiate into macrophages
Source: PLoS One. 2025 May 6;20(5):e0306150. doi: 10.1371/journal.pone.0306150 (PMC12054920; doi:10.1371/journal.pone.0306150)

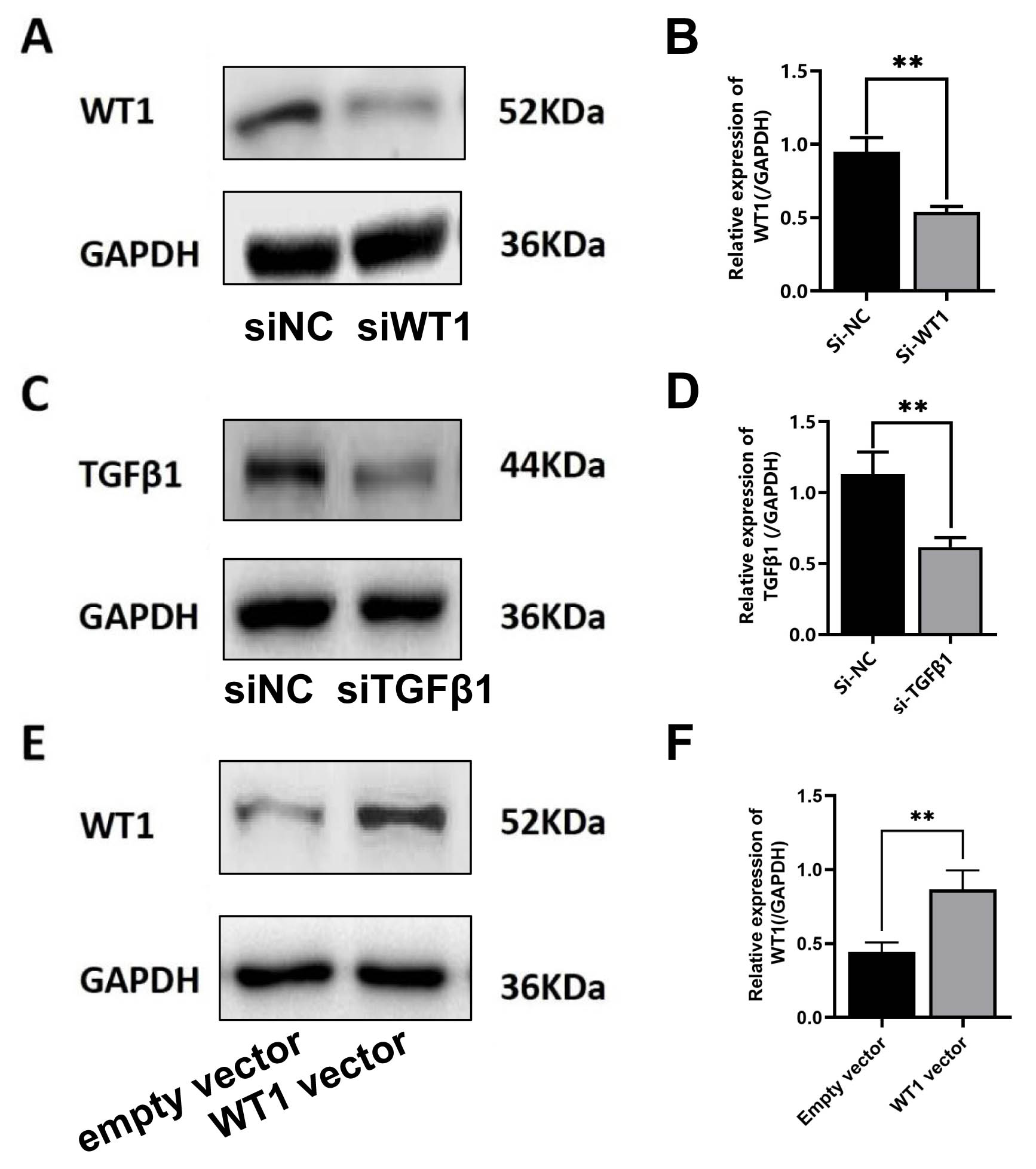

Supplement: S1 Fig — A. The WT1 expression by Western blotting assay in THP-1 cells transfected with siNC and si-WT1; B. The mean relative expression of WT1 in THP-1 cells transfected with siNC and si-WT1; C. The TGF-β1 expression by Western blotting assay in THP-1 cells transfected with siNC and si-TGF-β1; D. The mean relative expression of TGF-β1 in THP-1 cells transfected with siNC and si-TGF-β1; E. The WT1 expression by Western blotting assay in THP-1 cells transfected with empty vector and WT1 vector; F. The mean relative expression of WT1 in THP-1 cells transfected with empty vector and WT1 vector. (TIF) [file pone.0306150.s001.tif]
