# Supplementary material for: The up-regulation of TGF-β1 by miRNA-132-3p/WT1 is involved in inducing leukemia cells to differentiate into macrophages
Source: PLoS One. 2025 May 6;20(5):e0306150. doi: 10.1371/journal.pone.0306150 (PMC12054920; doi:10.1371/journal.pone.0306150)
Supplement: Attachment — Submitted filename: revision letter.docx [file pone.0306150.s003.docx]

Dear editor

Thank you very much for your valuable suggestions and opinions once again. As what you suggested, we have provided the original results corresponding to all the charts. It is necessary for us to further explain the results of protein expression here. The protein expression by Western blotting we provided earlier were based on validation of various proteins by some pre-experiments. Therefore, not all experiments and control groups were simultaneously set with standard band markers. Currently, based on your suggestions and requirements, we have re-conducted protein assay for each group again. The results and trends are consistent with the results and conclusions provided earlier. Please refer to the new manuscript and related attachments for specific protein detection and changes in related data information. As to the Western blotting, in addition, the average grayscale level of the three experiments can eliminate the bias factor of the one-time experiment and accurately reflect the trend of protein expression changes.

Sincerely yours

Jiang
